# Drought and Epidemic Typhus, Central Mexico, 1655–1918

**DOI:** 10.3201/eid2003.131366

**Published:** 2014-03

**Authors:** Jordan N. Burns, Rudofo Acuna-Soto, David W. Stahle

**Affiliations:** University of Arkansas, Fayetteville, Arkansas, USA (J.N. Burns, D.W. Stahle);; Universidad Nacional Autonoma de Mexico, Mexico City, Mexico (R. Acuna-Soto)

**Keywords:** typhus, epidemics, Rickettsia prowazekii, bacteria, body lice, Pediculus humanus corporis, drought, tree rings, Mexico

## Abstract

Epidemic typhus is an infectious disease caused by the bacterium *Rickettsia prowazekii* and transmitted by body lice (*Pediculus humanus corporis*). This disease occurs where conditions are crowded and unsanitary. This disease accompanied war, famine, and poverty for centuries. Historical and proxy climate data indicate that drought was a major factor in the development of typhus epidemics in Mexico during 1655–1918. Evidence was found for 22 large typhus epidemics in central Mexico, and tree-ring chronologies were used to reconstruct moisture levels over central Mexico for the past 500 years. Below-average tree growth, reconstructed drought, and low crop yields occurred during 19 of these 22 typhus epidemics. Historical documents describe how drought created large numbers of environmental refugees that fled the famine-stricken countryside for food relief in towns. These refugees often ended up in improvised shelters in which crowding encouraged conditions necessary for spread of typhus.

Epidemic typhus is a serious infectious disease caused by the obligate, intracellular, gram-negative bacterium *Rickettsia prowazekii* and transmitted by body lice (*Pediculus humanus* *corporis*). This disease is recognized for its high mortality rate throughout human history, particularly before modern sanitary practices and the availability of antimicrobial drugs (*1*). Typhus spreads where conditions are crowded and unsanitary. Historical epidemics often followed war, climate extremes, famine, and social upheaval. Zinsser (*2*) noted that throughout history, epidemic typhus might have claimed more human lives during war than combat. Epidemic typhus could reemerge as a serious infectious disease in areas of the world where social strife and underdeveloped public health programs persist (*3*).

Typhus was first recorded in Mexico in 1655, and the most recent major epidemic began in 1915 during the Mexican revolution. Mexico’s rich historical record of epidemic disease is documented in archives of demographic data that include census records, health records, death certificates, and accounts of physicians. Mexico City and the high, densely populated valleys of central Mexico were particularly susceptible to smallpox, cholera, and typhus epidemics because of crowding and poor sanitation (*4*). Numerous epidemics, some identified as typhus, occurred during the colonial and early modern eras. We have compiled a record of 22 typhus epidemics in Mexico during 1655–1918. We compared the timing of these typhus epidemics with tree-ring reconstructions of growing-season moisture conditions to assess the relationship between climate and typhus during this period.

## Background

The historical and geographic origins of typhus are disputed. Despite early evidence for typhus in Europe, it is unclear whether typhus was imported from Europe to the New World during colonization or vice versa (*3*). During the 16th century, typhus fever was gradually distinguished from diseases with similar clinical manifestations as physicians learned to recognize typhus by its sudden onset and characteristic rash (*5*). Epidemic typhus was not precisely distinguished from typhus fever until 1836 (*6*). Tabardillo, or Mexican typhus fever, was considered a disease separate from epidemic typhus until Dr. Howard Ricketts and others in 1910 proved that the 2 diseases were identical (*5*).

Epidemic typhus remains a threat in the rural highlands of South America, Africa, and Asia. Areas of Russia, Burundi, Algeria, and Andean Peru have all experienced typhus outbreaks in the past 20 years and are currently susceptible to outbreaks because of a high incidence of body lice, homelessness, or a large population of typhus survivors who are at risk for Brill-Zinsser disease (*6**–**9*). The risk for epidemic typhus has not been eliminated from more industrialized regions because body lice infestation still occurs in homeless populations in the United States, Europe, and the Netherlands (*10*).

Body lice infestation and typhus posed public health problems in Mexico until fairly recently. Cases of louse-borne typhus occurred mainly in cold months and in rural communities. From the late 19th century until 1963, the annual mortality rate of epidemic typhus in the rural state of Mexico decreased steadily from 52.4 to 0.1 cases/100,000 persons, and by 1979 no cases had been reported for 10 years. At the beginning of a 1980s program combating lice infestation, the rate of infestation with *P. humanus corporis* lice in the indigenous population of the state of Mexico was 100%. This rate decreased to 5%–12% by 1999. A 2002 study sampled the seroprevalence of typhus antibodies in ≈400 persons from communities in the state of Mexico. The prevalence of typhus antibodies in persons >65 years of age was 48%, and 6 case-patients who had particularly high levels of antibodies indicated possible Brill-Zinsser disease. Typhus survivors in Mexico are at risk for relapsing typhus fever and are potential sources for typhus outbreaks. An outbreak in Atlacomulco in 1967 was traced to a 76-year-old man with Brill-Zinsser disease (*11*).

## Data Sources and Methods

A total of 22 typhus epidemics during 1655–1918 were identified from historical records of disease in Mexico. Historical documentation of typhus epidemics includes almanacs; diaries; personal accounts; and medical and death records from hospitals, physicians, cemeteries, and municipalities. The terms typhus, symptomatic typhus, and tabardillo used in historical references were taken to mean epidemic typhus. When monthly mortality rate data were available, the onset of an epidemic was identified by an increase in the number of deaths from tens to hundreds over a 2-month period and by multiple historical references to typhus cases. Sources that indicated urgency; desperation; and a widespread need for supplies, medical care, hospitals, and cemeteries help distinguish normal typhus incidence from a typhus epidemic for which detailed mortality rate data were not available. A detailed summary of historical sources, including references, quotations, and a description for each epidemic, has been reported (J.N. Burns, unpub. data, http://cavern.uark.edu/misc/dendro/2013-Burns-Typhus.pdf).

**Figure 1:**
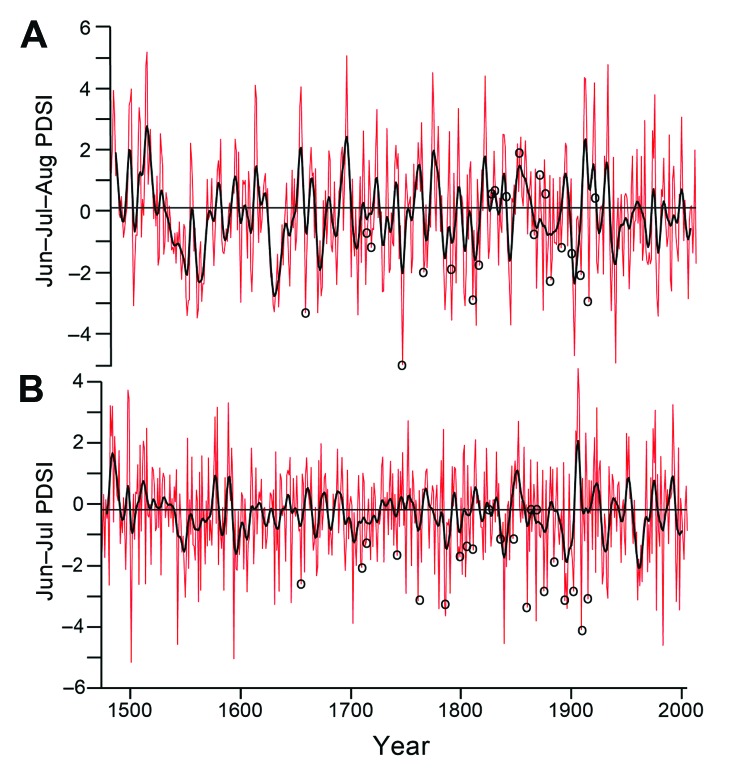
A) Time series of summer Palmer Drought Severity Index (PDSI) averaged for 78 grid points in central Mexico, 1665–1918. Data were obtained from Cook et al. (*12**,**13*) and Therrell et al. (*14*). B) Time series of June–July PDSI reconstructed from the Cuahtemoc la Fragua tree-ring chronology in east-central Mexico by using an average of 22 grid locations from the monthly PDSI dataset of R.R. Heim, Jr. (National Climatic Data Center, Ashville, NC, USA). Circles indicate typhus epidemics. Red lines indicate high-frequency yearly variability of moisture reconstruction. Black lines indicate smoothed lower-frequency representation of this variability. Horizontal lines indicate average PDSI for period.

The 22 identified typhus epidemics were compared with tree-ring–reconstructed soil moisture estimates for central Mexico by using 2 methods (Figure 1). The Palmer Drought Severity Index (PDSI) is used to represent the soil moisture balance and is computed from precipitation and temperature measurements incorporated into a numerical 2-layer soil moisture model (*12**,**15*). Station-based observations of monthly temperature and precipitation were mapped on a 0.5° latitude/longitude grid and then used to calculate monthly PDSI values for each grid point (R.R. Heim, Jr., National Climatic Data Center, Ashville, NC, USA) by using climatically aided interpolation techniques (*16*). A tree-ring reconstructed PDSI value for central Mexico is shown in Figure 1, panel A and a reconstructed PDSI value for east-central Mexico is shown in Figure 1, panel B. Years with mild, moderate, severe, and extreme droughts are indicated by PDSI values of −1, −2, −3, and −4, respectively (J.N. Burns, unpub. data). The corresponding wet conditions are indicated by PDSI values of +1, +2, +3, and +4. The years or periods in which typhus epidemics occurred are specified on each time series.

The reconstruction for central Mexico (Figure 1, panel A) was based on the gridded reconstructions of PDSI values for summers of the past millennium, which were compiled from >1,400 tree-ring chronologies developed across North America, including a few from northern and central Mexico (*15*). We extracted the gridded PDSI value reconstructions for central Mexico (78 grid points) and averaged the yearly estimates of each grid point into a single 500-year time series of summer PDSI value measurements for that region (summer PDSI values = June, July, August averaged PDSI values). Selected grid locations and reconstructed PDSI values for central Mexico have been reported (Burns JN, unpub. data). This time series approximates a history of drought and wetness over central Mexico.

A second reconstruction of PDSI values was made for east-central Mexico by using the earlywood width chronology derived from ancient Douglas fir trees at Cuauhtemoc la Fragua, Puebla (Figure 1, panel B). The Cuauhtemoc collection includes 205 radii from 85 different trees and fallen logs. Each tree ring was exactly dated by using standard dendrochronologic methods described by Douglass (*17*), and earlywood and latewood growth increments were measured by using a stage micrometer. The resultant earlywood width chronology represents a record of drought and wetness in east-central Mexico during 1474–2001. The Cuauhtemoc la Fragua chronology might be the most climate-sensitive long chronology developed for central Mexico and it has been used to study the role of climatic extremes in famine, disease, and social upheaval throughout Mexican history (*14**,**18*).

The Cuauhtemoc earlywood chronology was used to reconstruct June–July PDSI values for east-central Mexico (i.e., the Sierra Madre Oriental and the Valley of Mexico). The reconstruction was calibrated with instrumental June–July PDSI values for east-central Mexico by using bivariate regression for 1975–2001 (*R*^2^_adj_ = 0.54, based on an average of 22 grid points). This June–July PDSI value reconstruction verifies only weakly when compared with independent instrumental PDSI values for 1950–1974 (*r* = 0.3), and the reduction of error and coefficient of efficiency statistics are barely positive (0.003 and 0.003, respectively) (*19*). These marginal verification statistics suggest that the area of the June–July PDSI signal in east-central Mexico recorded by the single tree-ring chronology at Cuauhtemoc la Fragua has varied in size during the instrumental era. Nevertheless, the Cuauhtemoc la Fragua Douglas fir chronology has a strong moisture signal for the past 500 years in spite of the weak verification statistics (*14**,**18*).

**Figure 2:**
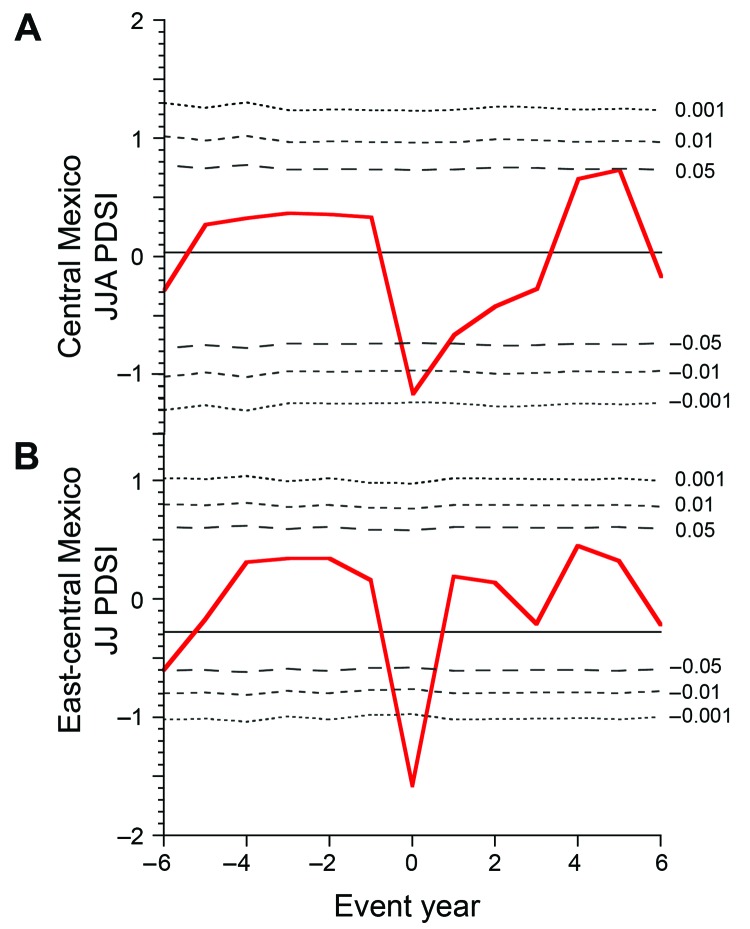
A) Superposed epoch analysis (*20*) of summer Palmer Drought Severity Index (PDSI) for central Mexico averaged for the 22 periods that had typhus epidemics (1655, 1710–1712, 1714, 1742, 1761–1762, 1785–1787, 1799–1802, 1805–1806, 1811–1812, 1821–1823, 1825–1828, 1835–1838, 1847–1848, 1861–1864, 1865–1868, 1870–1873, 1875–1877, 1884–1886, 1894–1895, 1902–1903, 1909–1911, and 1915–1918). Horizontal line indicates PDSI = 0. JJ, June–July; JJA, June–July–August. B) Superimposed epoch analysis of June–July PDSI for east-central Mexico also averaged for the 22 periods that had typhus epidemics. Superimposed epoch analyses were performed by using the Dendrochronology Program Library (*21**,**22*). Horizontal line indicates mean PDSI.

The statistical significance of the relationship between drought and epidemic typhus in Mexico during 1655–1918 was assessed by using superposed epoch analysis (*20*). Reconstructed PDSI values were sorted and averaged for years before, during, and after the 22 historical typhus epidemics. The mean PDSI value was computed for the typhus event years or periods and was then tested against the mean of all other years in the reconstructed time series for central (Figure 2, panel A) and east-central Mexico (Figure 2, panel B). The means for each of 6 years before and after each epidemic was also tested against the mean of all other years to examine any cumulative or lag effects of drought on the typhus epidemics.

**Figure 3:**
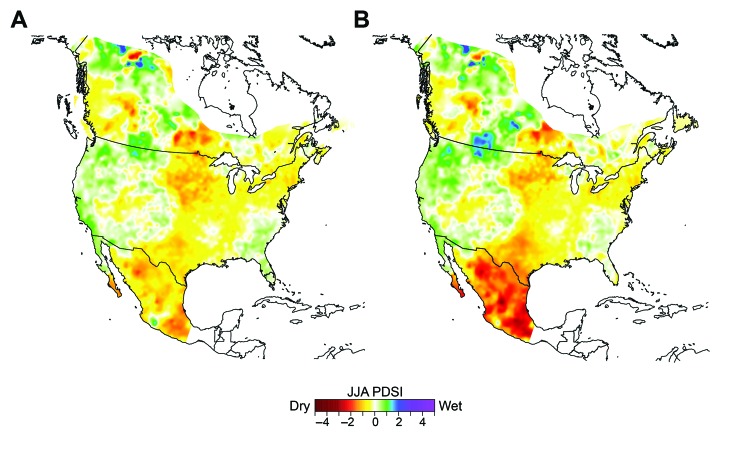
Tree-ring–reconstructed summer Palmer Drought Severity Index (PDSI) during A) 22 and B) 15 typhus epidemics in Mexico, 1665–1918. Drought conditions are indicated by negative values on the PDSI scale. Reconstructed summer PDSI values during the 15 typhus epidemic years with the most negative PDSI values for central Mexico are mapped in panel B. JJA, June–July–August.

The reconstructed summer PDSI values for all epidemic years or periods were averaged at each of the 11,396 continent-wide grid points, and these averages were mapped to represent the spatial anomaly of growing season moisture during the catastrophes; an updated version (*13*) of the reconstructions of Cook et al. (*12*) was used. The reconstructed PDSI values for all typhus epidemic years are mapped in Figure 3, panel A, and the PSDI values for the 15 driest epidemic years are mapped in Figure 3, panel B to highlight the area of Mexico most afflicted by drought conditions during these epidemics.

## Historical Typhus Epidemics

Historical citations from Mexico during 1655–1918 leave no doubt that drought and famine were associated with some serious epidemics of typhus. In fact, drought, famine, and/or crop failure was reported by historical sources during 15 of the 22 periods of epidemic typhus and was described as markedly severe or widespread during the epidemics of 1714, 1785, 1861, 1875, 1884, 1909, and 1915 (J.N. Burns, unpub. data). The tone used in historical references concerning these years suggests a state of national or regional emergency, whereas references from less severe epidemics suggest that the outbreaks were localized crises. Drought and a severe early frost coincided during 1785, sharply reducing harvests and causing a famine so widespread that 1785 became “El Año del Hambre” (“the year of hunger”) in Mexican history (*18**,**23*). This year of starvation coincided with the typhus epidemic of 1785. Again, from 1909 through 1911, epidemic typhus coincided with severe frost and a corn crop failure so massive that corn import taxes were eliminated. Concise descriptions of each of the 22 epidemics were synthesized from dozens of historical citations from Mexico and were compiled (J.N. Burns, unpub. data).

Information from historical sources about past climate and typhus epidemics is variable because reports come from many authors and they do not describe events consistently, especially environmental conditions. A more consistent and independent proxy for moisture variability was needed to test the possible role of climate in past typhus outbreaks. We used tree rings because they can be exactly dated and are well correlated with available soil moisture data.

## Tree Ring–Reconstructed Drought

When the 22 typhus epidemics were compared with tree-ring reconstructions of summer PDSI values, we noted below average tree growth and reconstructed drought during the onset year of most typhus epidemics (15 of 22 typhus periods in central Mexico (Figure 1, panel A). Many of these epidemics occurred during years when reconstructed moisture for central Mexico was far below average (Figure 1, panel A). The time series for east-central Mexico indicates that 19 of the 22 epidemics occurred during drought and that only 3 typhus events occurred during years with near-normal reconstructed moisture estimates (Figure 1, panel B).

The superposed epoch analyses indicate that the reconstructed mean PDSI values for all 22 epidemic periods in central Mexico was significantly below the average of all remaining years (mean −1.16; p<0.01) (Figure 2, panel A). This drought pattern was especially pronounced in the reconstructed PDSI values for east-central Mexico during the 22 events (mean −1.68; p<0.001) (Figure 2, panel B). These results suggest that drought may have been a major factor in the spread of typhus in colonial and early modern Mexico (Figure 2, panel A).

The continent-wide reconstructed PDSI values mapped in Figure 3, panel A, indicate mild drought conditions over most of Mexico during the 22 periods in which typhus epidemics occurred. When the 7 epidemic years with near-normal to above-average moisture reconstructions are omitted from the mapping of reconstructed PDSI values, drought for the remaining 15 epidemic years appears much more intense, particularly over east-central Mexico (Figure 3, panel B). This finding suggests that the most severe drought during those 15 typhus epidemics might have been located in east-central Mexico, where most of the 22 typhus epidemics were concentrated, according to historical documentation (J.N. Burns, unpub. data).

## Discussion

The observed relationship between drought and typhus epidemics in colonial and modern Mexico is curious because drought has not been specifically considered as a risk factor for typhus. Nevertheless, drought, much like war and natural disaster, has caused famine in poor agricultural regions and can promote migration of impoverished refugees from the countryside into city centers in search of food and relief (*24**,**25*). An influx of starving persons without money, jobs, or shelter into urban areas in Mexico might have amplified the crowded and unsanitary conditions necessary for historical outbreaks of epidemic typhus.

There is some support for this environmental refugee theory in historical documents from Mexico, in which drought-induced famines and influxes of poor persons into cities were sometimes recorded. For example, a wealthy man described the city of Guanajuato during the typhus epidemic of 1714, when crowds of poor persons gathered in the street to beg for food: “Walking on the streets it was common to find people reduced almost to their bare skeletons walking in bands. With almost no strength they kneeled on front of you and said in a very faint voice ‘For God’s love, we are dying of starvation, help us in our necessity, you powerful man.’ It was painful to see the poor and sick who, because of their great necessity, came out to the streets with all their bodies shaking, surely a bad thing for their disease” (J.N. Burns, unpub. data).

Another account described the typhus epidemic of 1785, which coincided with the year of hunger in city of Queretaro: “The epidemic competes with famine for the number of deaths. In the mountains, hills, streets, was a theater of the saddest spectacle, long caravans of miserable people walked in all directions asking, for God’s love, a piece of bread. Very often some of them fell and died shortly after.” During this time, according to Cooper (*4*), “concurrently the region suffered ‘a series of natural calamities which completely destroyed the grain crop and reduced large sections of the population to a state of extreme poverty and famine. That winter [of 1785] and the spring of 1786 saw thousands of desperate farmers and workmen roaming the countryside, swarming into the towns in search of food, and dying of starvation and disease’” (J.N. Burns, unpub. data). 

The epidemic of 1861 coincided with drought and high grain prices in central Mexico. Historical sources agree that the epidemic was triggered by the mass movement of soldiers and starving refugees into Mexico City in search of food and relief. During the 1875–1877 epidemic, all of Mexico experienced severe food scarcity while a large wave of poor immigrants arrived in Mexico City. Mass migration of refugees into cities, spurred by food scarcity and the ongoing revolution, also may have played a role in the epidemic of 1915–1918 (J.N. Burns, unpub. data).

## Conclusions

Substantial evidence has been found for the role of drought in epidemic typhus during 1655–1918 in Mexico. The historical records implicate environmental refugees as the possible mechanism by which drought contributed to the spread of epidemic typhus during the 22 examined epidemics. However, this theory needs further investigation. Verification of the role of environmental refugees in the transmission of typhus during these 22 epidemics is complicated by a lack of clarity in the historical sources and by the interaction between politics, geography, and climate. The exact timing for the onset and withdrawal of drought, famine, or typhus epidemics as recorded in historical sources can be ambiguous. Gaps and contradictions in the historical records need to be resolved through a more detailed, multifocal historical analysis. In addition, most historical citations describing typhus outbreaks are from Mexico City, but tree-ring reconstructions indicate that drought was much more widespread in Mexico during these events. The true spatial extent of typhus during these 22 and perhaps other epidemics needs to be identified in the historical records of other localities.

The long-term collections of historical data have provided insight into epidemics of typhus during the era before antimicrobial drugs. Detailed chronology of typhus epidemics and tree-ring data are rich and unexhausted scientific resources but, unfortunately, both are threatened by inadvertent destruction by archival destruction and deforestation (*26*). Mexico has a long and detailed historical record and some of the utmost forest biodiversity in the world. The integration of historical epidemiologic data with proxy climate data from tree rings promises to improve understanding of the interactions between climate and society that result in epidemic disease in Mexico.
